# Screening of Natural Stilbene Oligomers from *Vitis vinifera* for Anticancer Activity on Human Hepatocellular Carcinoma Cells

**DOI:** 10.3390/antiox9060469

**Published:** 2020-06-01

**Authors:** Iris Aja, M. Begoña Ruiz-Larrea, Arnaud Courtois, Stéphanie Krisa, Tristan Richard, José-Ignacio Ruiz-Sanz

**Affiliations:** 1 Free Radicals and Oxidative Stress (FROS) research group of the Department of Physiology, Medicine and Nursing School, University of the Basque Country UPV/EHU, 48940 Leioa, Spain; iris.aja@ehu.eus (I.A.); joseignacio.ruizs@ehu.eus (J.-I.R.-S.); 2 Univ. Bordeaux, INRAE, UR Œnologie, EA 4577, USC 1366, ISVV, 210 Chemin de Leysotte, F 33882 Villenave d’Ornon, France; arnaud.courtois@u-bordeaux.fr (A.C.); stephanie.krisa@u-bordeaux.fr (S.K.); tristan.richard@u-bordeaux.fr (T.R.)

**Keywords:** stilbene, resveratrol oligomers, natural product, polyphenol, anticancer activity, HepG2 cells

## Abstract

The characterization of bioactive resveratrol oligomers extracted from *Vitis vinifera* canes has been recently reported. Here, we screened six of these compounds (ampelopsin A, *trans-*ε-viniferin, hopeaphenol, isohopeaphenol, R2-viniferin, and R-viniferin) for their cytotoxic activity to human hepatocellular carcinoma (HCC) cell lines p53 wild-type HepG2 and p53-null Hep3B. The cytotoxic efficacy depended on the cell line. R2-viniferin was the most toxic stilbene in HepG2, with inhibitory concentration 50 (IC_50_) of 9.7 ± 0.4 µM at 72 h, 3-fold lower than for resveratrol, while Hep3B was less sensitive (IC_50_ of 47.8 ± 2.8 µM). By contrast, hopeaphenol (IC_50_ of 13.1 ± 4.1 µM) and isohopeaphenol (IC_50_ of 26.0 ± 3.0 µM) were more toxic to Hep3B. Due to these results, and because it did not exert a large cytotoxicity in HH4 non-transformed hepatocytes, R2-viniferin was selected to investigate its mechanism of action in HepG2. The stilbene tended to arrest cell cycle at G2/M, and it also increased intracellular reactive oxygen species (ROS), caspase 3 activity, and the ratio of Bax/Bcl-2 proteins, indicative of apoptosis. The distinctive toxicity of R2-viniferin on HepG2 encourages research into the underlying mechanism to develop the oligostilbene as a therapeutic agent against HCC with a particular genetic background.

## 1. Introduction

Hepatocellular carcinoma (HCC) is the most frequently diagnosed primary cancer of the liver [1] and the fourth most common cause of cancer-related death worldwide [2]. There is currently no effective treatment for HCC due to the high heterogeneity of the cancer. Thus, extrinsic factors and different mutations contribute to the induction of liver cancer [3,4]. Among the genetic factors, mutations in the p53 tumor suppressor gene (TP53) are frequent, especially in HCC from populations exposed to environmental carcinogens, such as dietary aflatoxin B1 [5]. In these cases, anti-tumor strategies include p53 activation. However, in a high proportion of hepatocarcinomas, p53 is retained, and recently, it has been proven that depending on the specific isoform it could also have an oncogenic role. Thus, the specific p53 family isoform is the target of novel anticancer therapies against HCC [6].

Several tumor cell lines with different phenotypes that resemble the various types of liver cancers have been used. These approaches are valid and allow the results to be extrapolated to an in vivo situation [7].

Since the discovery of the natural antitumor drug paclitaxel in 1960s, the interest in identifying new natural chemotherapeutic agents, particularly those with phenolic structure, has greatly increased. Stilbenes are a class of polyphenols characterized by the presence of a 1,2-diphenylethylene moiety. They are produced as plant secondary metabolites and exert protective actions against environmental challenge, acting mainly as antifungal phytoalexins [8]. Stilbenes are present in foods and beverages such as blueberries, peanuts, grapes, and red wine [9]. Grapevine is one of the richest sources of stilbenes currently known. The cane and grape stems and seeds show high concentrations of stilbenes [10,11], which are also present in grape skin and juice and in red wines [12]. Resveratrol is the most widely studied stilbene for its actions on human health. Different works have reported its properties against diseases such as diabetes [13], cancer [14], cardiovascular diseases [15], and neurodegenerative diseases [16]. However, in the biosynthetic pathway, as the result of various oxidative condensations of resveratrol monomer, several dimers, trimers and tetramers are formed. Despite the numerous works describing the beneficial effects of resveratrol on health, other natural stilbenes, particularly oligomers, have received far less attention. The wine industry generates a high quantity of waste (wood, cane, and root) and oligostilbens are the main stilbenes extracted from these wastes, which constitute a cheap source of bioactive products [11]. In this work, we have studied the cytotoxic potential of a range of resveratrol oligomers (dimers and tetramers), extracted and purified from the *Vitis vinifera* grapevine cane, on hepatoma cell lines. The effects were compared with those of resveratrol. After selecting the most active compound, we investigated the mechanism of its action.

## 2. Materials and Methods

### 2.1. Stilbenes from Vitis vinifera

Ampelopsin A, *trans-*ε-viniferin, hopeaphenol, isohopeaphenol, R2-viniferin, and R-viniferin were obtained from extracts of grapevine cane (*Vitis vinifera*), named Vineatrol^®^30. The extract was kindly provided by Actichem S.A. (Montauban, France). The isolation, characterization and purification of the stilbenes were carried out as described previously [17]. The purity of the compounds was estimated to be ≥95%. *trans*-Resveratrol was purchased from Sigma-Aldrich (Lyon, France).

### 2.2. Cell Culture

The human hepatoma cell line HepG2 was obtained from the American Tissue Culture Collection (ATCC, Manassas, VA, USA). The human hepatoma cell line Hep3B was obtained from the European Collection of Authenticated Cell Cultures (ECACC, Porton Down, Salisbury, Wiltshire, UK). HH4 non-transformed human hepatocyte cell line (kindly provided by Dr. I. Fabregat, Molecular Oncology, Bellvitge Biomedical Research Institute, Spain) was established as described [18].

HepG2 and Hep3B cell lines were maintained in Eagle’s Minimum Essential Medium and HH4 cell line was grown in Ham´s F12 Nutrient Mixture (Sigma-Aldrich, St Louis, MO, USA). Both culture media were supplemented with 10% heat inactivated fetal bovine serum, 2 mM L-glutamine and antibiotics (0.1 mg/mL streptomycin and 100 U/mL penicillin) (Sigma-Aldrich, St Louis, MO, USA). Cell cultures were grown in an incubator at 37 °C with 5% CO_2_ atmosphere. After reaching approximately 80% of confluence, cells were detached in a solution of 0.1% trypsin and 0.04% EDTA and plated as required for further experiments.

### 2.3. Cell Viability Assay

Cell lines were seeded into 96-well plates at 5 × 10^3^ (HepG2) and 3 × 10^3^ (Hep3B and HH4) cells/well 24 h before treatment. Increasing concentrations of the monomer *trans*-resveratrol, the dimers ampelopsin A and *trans*-ε-viniferin and the tetramers isohopeaphenol, hopeaphenol, R2-viniferin and R-viniferin were then added and cells were incubated for 24, 48, and 72 h. Stilbenes were dissolved in dimethyl sulfoxide (DMSO), Sigma-Aldrich, St Louis, MO, USA) at a final concentration of 0.01%. The same amount of DMSO was added to control cells. After treatment, the cell viability was determined using the crystal violet assay [18]. The absorbance was recorded at 590 nm in a Synergy HT microplate reader (BioTek, Winooski, VT, USA). Cell viability was calculated as the percentage of viable cells treated with stilbene versus untreated control cells using the following equation: Cell viability (%) = [OD (Treatment) − OD (Blank)]/[OD (Control) − OD (Blank)] × 100. The cytotoxic effect of stilbenes was determined by calculating IC_50_ values using non-linear regression analysis (GraphPad Prism 6, San Diego, CA, USA).

### 2.4. Cell Cycle Analysis

Exponentially growing cells were seeded in 6-wells plates (200,000 cells HepG2 and 180,000 cells Hep3B) 24 h before R2-viniferin treatment. On the day of treatment, the culture medium was replaced with culture medium containing R2-viniferin or vehicle solution (control).

After a treatment, cells were detached by trypsinization and fixed overnight at 4 °C in 70% ice-cold ethanol diluted in phosphate buffer saline (PBS). The ethanol was then discarded and the cells were washed with ice-cold PBS and finally stained with 25 μg/mL of propidium iodide (Sigma-Aldrich, St Louis, MO, USA) in the presence of 200 μg/mL ribonuclease A (Roche Biochemicals, Indianapolis, IN, USA) for 30 min at room temperature in the dark. Flow cytometry analysis (Beckman Coulter Gallios) was carried out from a total number of 10,000 events acquired in the General Research Services SGIker of the UPV/EHU. Analysis of the data was performed using Summit 4.3 software (Dako, Glostrup, Hovedstaden, Denmark).

### 2.5. Intracellular ROS and Mitochondrial O_2_^−^ Measurement

Intracellular levels of reactive oxygen species (ROS) were measured using the cell-permeant 2’,7’ dichlorodihydrofluorescein diacetate (H_2_DCF-DA) probe (Molecular Probes, Eugene, OR, USA), which permeates living cells and is deacetylated and oxidized upon ROS exposure inside the cell, forming the fluorescent 2’,7’-dichlorofluorescein (DCF).

The mitochondrial superoxide anion (O_2_^−^) levels were measured using MitoSOX™Red reagent (Molecular Probes, Eugene, OR, USA), which permeates living cells where it selectively targets mitochondria and is oxidized by superoxide.

HepG2 cells were cultured in a 6-well cell culture plate at a density of 2 × 10^5^ cells per well 24 h before starting the treatment with R2-viniferin. The medium was renewed and cells were incubated with R2-viniferin as described above. After treatment, cells were washed and incubated with 20 μM H_2_DCF-DA or 4 μM MitoSOX™ for 30 min at 37 °C in the dark. The probe solution was then withdrawn and, after washing with PBS, the cells were trypsinized. The fluorescence intensity from living cells was measured by flow cytometry (DCF λex = 485/20 and λem = 528/20, MitoSOX λex = 485/20 and λem = 620/20) in a Beckman Coulter Gallios Flow cytometer in the General Research Services SGIker of the UPV/EHU. At least 10,000 events were detected. Data obtained from flow cytometry were analyzed using Summit 4.3 software (Dako, Hovedstaden, Denmark). Intracellular ROS and mitochondrial O_2_^−^ were expressed as the percentage of the fluorescence intensity in control cells at the same time of incubation.

### 2.6. Caspase-3 Activity

The activity of caspase-3 was measured using the specific synthetic tetrapeptide fluorogenic substrate Ac-DEVD-AMC (BD Pharmigen Biosciences, San Diego, CA, USA). The assay was carried out in 96-well plates in a total volume of 100 µL, with 37 μM of the substrate and 50 µg of protein in the assay medium in 100 mM HEPES, pH 7.4, containing 200 mM NaCl, 0.2% CHAPS, 2 mM EDTA, 20% glycerol, and 5 mM dithiothreitol (DTT).

In the presence of caspase-3, the Ac-DEVD-AMC substrate is hydrolyzed and the fluorogenic compound AMC is released. The activity was determined by continuous recording of the fluorescence at λex = 360 nm and λem = 460 nm at 37 °C for 2 h every 5 min in a Synergy HT microplate reader (BioTek, Winooski, VT, USA). Results were expressed as the percentage of the fluorescence in control cells.

### 2.7. Western Blot Analysis

HepG2 cells were seeded in Petri dishes, incubated for 24 h and then treated with 5 and 10 µM R2-viniferin. Following 24 h of treatment, the cells were lysed in ice for 30 min with lysis buffer (20 mM HEPES pH 7.5, 1 mM NaF, 10 mM EGTA, 40 mM β-glycerophosphate, 1% NP-40, 2.5 mM MgCl_2_, 2 mM orthovanadate, and 1 mM DTT) to which 10 μL/mL of a protease inhibitor cocktail (Sigma-Aldrich, St Louis, USA) was added just before use. Cellular fragments were removed by centrifugation at 12,000× *g* for 10 min at 4 °C, and total protein concentration was determined by Bradford assay [19]. The cellular protein extracts were boiled at 95 °C for 5 min in Laemmli buffer and separated by sodium dodecyl sulfate–polyacrylamide gel electrophoresis in 15% polyacrylamide gels. Proteins were transferred onto polyvinylidene difluoride (PVDF) membranes by electro-blotting with constant amperage (1 mA/cm^2^) for 2 h in a wet chamber. After blocking for 1 h in 5% bovine serum albumin (BSA) Tris-buffered saline containing Tween 20 at room temperature, membranes were incubated overnight at 4 °C with antibodies to Bax (Santa Cruz Biotechnology, 1:500), Bcl-2 (Santa Cruz Biotechnology, 1:500) and glyceraldehyde-3-phosphate dehydrogenase (GAPDH, Abcam, 1:2000). After washing, membranes were probed with their corresponding secondary antibody conjugated to horseradish peroxidase for 1 h at room temperature. Specific proteins were detected using the enhanced chemiluminescence (ECL) substrate kit (Clarity Western ECL substrate, Bio-Rad, Hercules, California, USA) and the blots were imaged with the C-DiGit LI-COR blot scanner (Bonsai Advanced technologies S.L. Madrid, Spain). Intensities of target protein bands were determined by densitometry and normalized to the intensity of the loading control GAPDH protein.

### 2.8. Statistical Analysis

The statistical package SPSS 19.0 (SPSS Inc. Chicago, IL, USA) was used for data analysis. The results were expressed as mean ± standard error (SE) from at least three experiments. Statistical significance for the differences of the means was estimated by parametric Student’s *t*-test. Differences between means were considered statistically significant for *p* < 0.05. IC_50_ values were derived from fitting the data to a sigmoidal dose-response curve with a three-parameter logistic model using Graph Pad Prism 6.

## 3. Results

**Figure 1:**
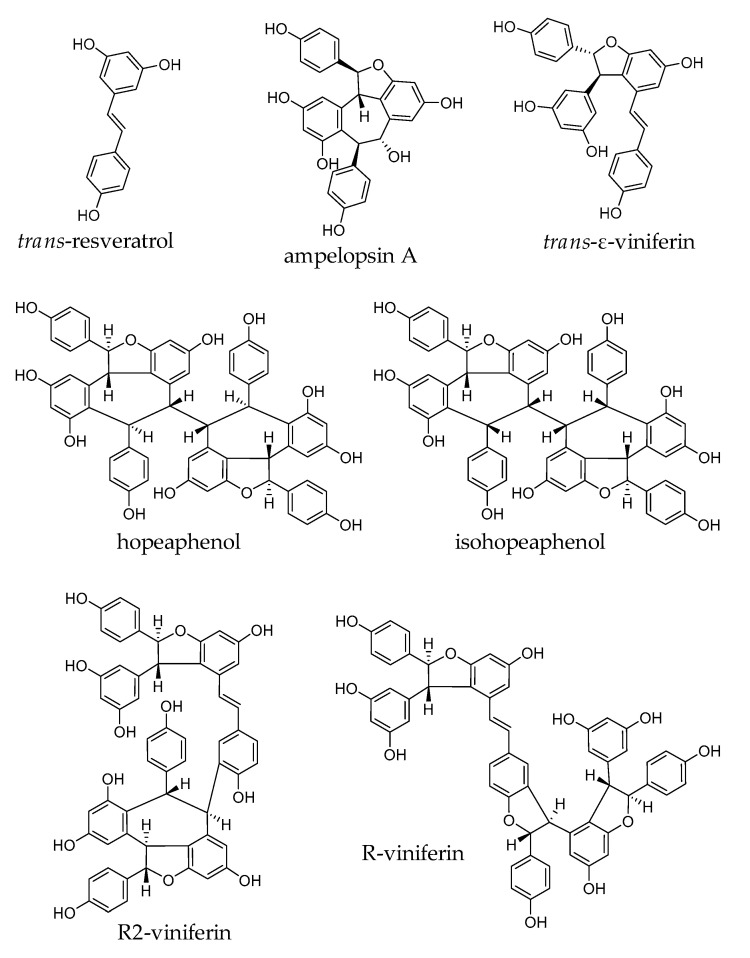
Chemical structures of resveratrol and resveratrol oligomers.

In this work, we have investigated the cytotoxicity of one stilbene monomer: *trans*-resveratrol; two stilbene dimers: ampelopsin A and *trans-*ε-viniferin; and four resveratrol tetramers: hopeaphenol, isohopeaphenol, R2-viniferin, and R-viniferin. Their chemical structures and that of resveratrol are shown in Figure 1.

**Figure 2:**
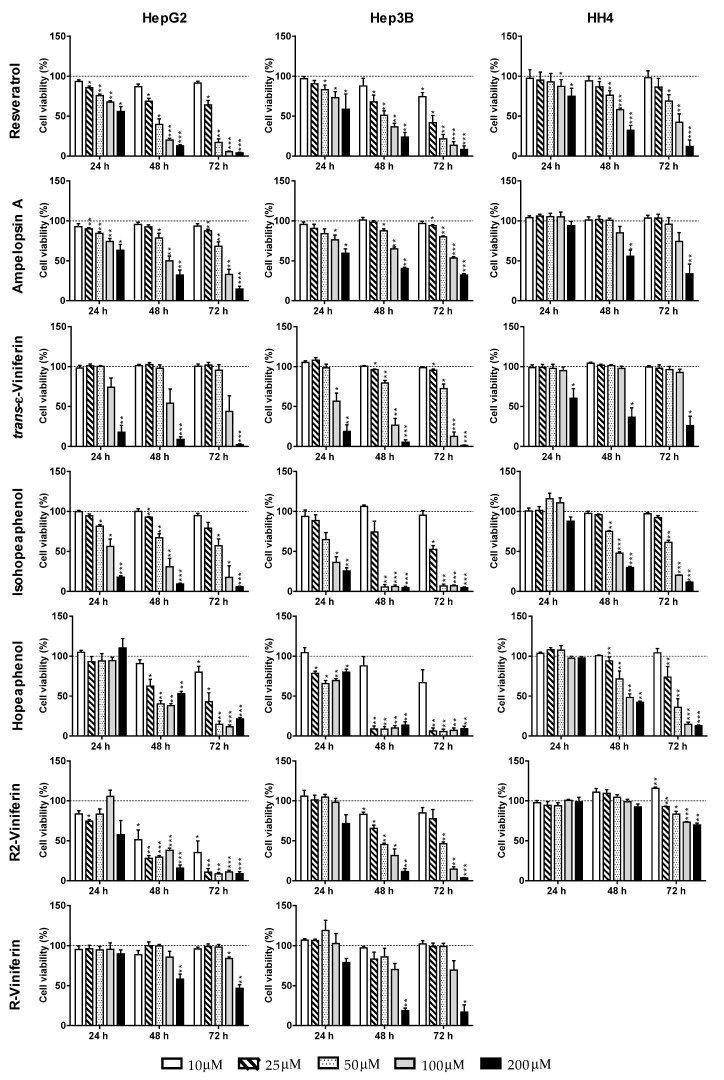
Effect of different stilbenoids on cell viability of HepG2, Hep3B and HH4. Cell viability was measured by crystal violet assay. Results are the mean + SE of *n* = 3–5 experiments. * *p* < 0.05, ** *p* < 0.01, *** *p* < 0.001, compared with control at the same time.

The cytotoxic activities of resveratrol oligomers were screened on HepG2, Hep3B and HH4. Cells were treated with increasing concentrations of each stilbene and after 24, 48, and 72 h, cell viability was measured by crystal violet assay (Figure 2).

IC_50_ values were calculated from the inhibition curves (Table 1). The stilbenes induced a decrease of cell number in a dose- and time- dependent manner. Resveratrol, the reference stilbene, reduced the viability of HepG2 in a similar way to Hep3B, with IC_50_ values of 30 µM (in HepG2) and 21 µM (in Hep3B) at 72 h. In non-transformed hepatocytes, the concentration needed to reduce cell viability by 50% was markedly higher (93 µM).

Among dimers, ampelopsin A was more active in HepG2 (IC_50_ of 76 µM), while *trans-*ε-viniferin was more cytotoxic in Hep3B (IC_50_ 63 µM). In non-transformed hepatocytes, much higher concentrations of stilbene dimers were needed to induce toxicity. Among tetramers, hopeaphenol and isohopeaphenol were highly cytotoxic to Hep3B, with effective IC_50_ at 72 h of 13 µM and 26 µM, respectively. Their effects in HepG2 were less pronounced, with hopeaphenol (IC_50_ of 24 µM) being twice as potent as its geometric isomer isohopeaphenol (54 µM). R2-viniferin was the most toxic compound among all tested stilbenes in HepG2. The IC_50_ value was < 10 µM, more than three times lower than that found for resveratrol. In Hep3B the stilbene was not so efficient, showing an IC_50_ of 48 µM. Interestingly, in HH4 non-transformed hepatocytes the IC_50_ value was higher than 200 µM (Table 1). In the case of R-viniferin, containing one less free hydroxyl group than R2-viniferin, concentrations as high as 200 µM were necessary to induce cell death in HepG2 and Hep3B (Figure 2).

**Figure 3:**
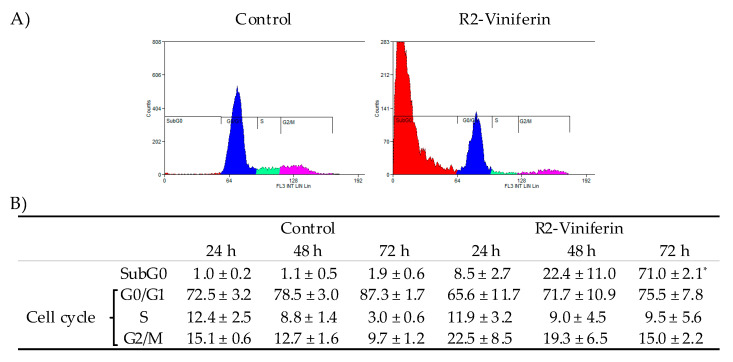
Cell cycle distribution of HepG2 treated with R2-viniferin. (**A**) Representative graphs of cell cycle analysis by flow cytometry at 72 h. (**B**) Statistical analysis of cell cycle distribution. Data are expressed as the percentage (%) of cells at different stages of cell cycle. In the case of cells in subG0, data are expressed as the percentage of total cells. Results are the mean ± SE of three experiments. * *p* < 0.001, significantly different from control.

**Figure 4:**
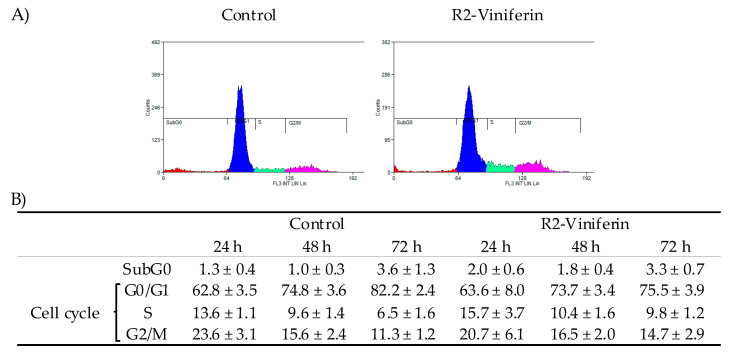
Cell cycle distribution of Hep3B treated with R2-viniferin. (**A**) Representative graphs of cell cycle analysis by flow cytometry at 72 h. (**B**) Statistical analysis of cell cycle distribution. Data are expressed as the percentage (%) of cells at different stages of cell cycle. In the case of cells in subG0, data are expressed as the percentage of total cells. Results are the mean ± SE of three experiments.

In view of these results, R2-viniferin was selected to study the mechanisms of its cytotoxicity in hepatocellular carcinoma cells. The effect of R2-viniferin on cell cycle distribution in both HepG2 and Hep3B was studied by flow cytometry. As can be seen in Figure 3, HepG2 treated with 10 µM R2-viniferin showed a progressive increase in the number of cells in subG0 phase over time. This effect was accompanied by a concomitant, though not significant, increase in the percentage of cells in the G2/M phase, suggesting an arrest in this phase. In Hep3B, however, R2-viniferin did not alter cell cycle at this concentration (Figure 4).

**Figure 5:**
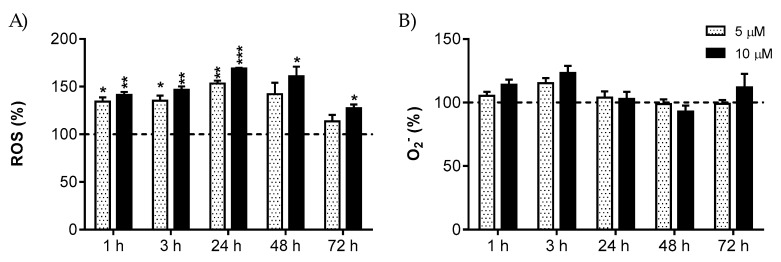
Effect of R2-viniferin on (**A**) intracellular ROS and (**B**) mitochondrial O_2_^−^ levels in HepG2. Cells were treated with 5 and 10 µM of R2-viniferin for the indicated times. Reactive species were detected by flow cytometry. (**A**) ROS were determined by H_2_DCF-DA assay. (**B**) O_2_^−^ was determined by MitoSOX probe. Results are expressed as the percentage (%) of the control values at the same time, and are the mean ± SE of 3 experiments. * *p* < 0.05; ** *p* < 0.01; *** *p* < 0.001.

R2-viniferin increased the intracellular ROS concentration dose-dependently from the first time assayed (Figure 5A). At the highest concentration used (10 µM), ROS remained significantly elevated up to 72 h. In the case of mitochondrial O_2_^−^, no significant effect could be observed at any of the stilbene doses or times assayed (Figure 5B).

**Figure 6:**
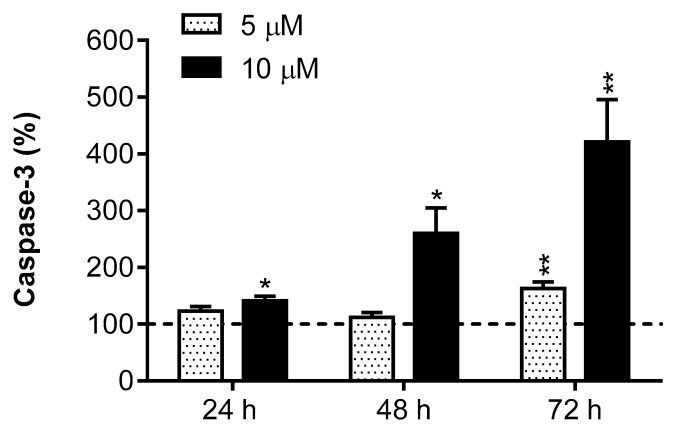
Effect of R2-viniferin on caspase-3 activity in HepG2. Cells were incubated with R2-viniferin (5 μM and 10 μM) at the indicated times. Results are expressed as the percentage (%) of the control values, and are the mean ± SE of 4 experiments. * *p* < 0.05, ** *p* < 0.01, compared with controls.

R2-viniferin (10 µM) increased the caspase-3 activity significantly from 24 h to 72 h. At 5 µM concentration the stilbene increased caspase-3 activity at 72 h (Figure 6). These data indicate that R2-viniferin induces HepG2 death through a caspase-dependent mechanism, in which the executioner caspase-3 is involved.

**Figure 7:**
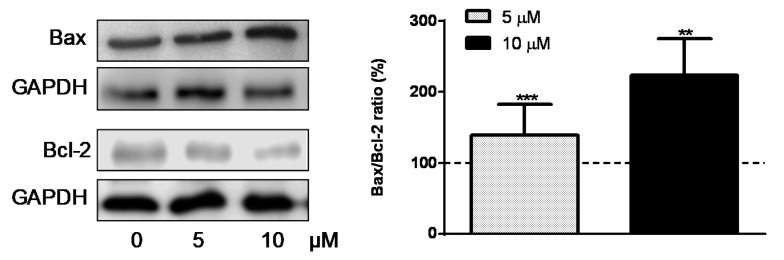
Western blot analysis of Bax and Bcl-2 expression in HepG2 cells incubated with R2-viniferin (5 and 10 µM). The bar graph shows the Bax/Bcl2 ratio, calculated by densitometric analysis of the anti-apoptotic proteins expressed as the percentage of the ratio (molecule of interest/GAPDH protein) detected in the control. Results are the mean + SE of *n* = 6 experiments. ** *p* < 0.01, *** *p* < 0.001, compared with control.

We then analyzed by the effect of R2-viniferin on the apoptosis-related Bcl-2 protein family. The resveratrol oligomer upregulated the expression of the proapoptotic Bax protein and downregulated anti-apoptotic Bcl-2 proteins (Figure 7). The Bax/Bcl-2 ratio increased dose-dependently (39% and 123%) over control at 5 µM and 10 µM, respectively.

## 4. Discussion

The results presented herein show for the first time that several natural oligomeric products of resveratrol, in particular tetramers, are cytotoxic to human hepatocellular carcinoma cells, inhibiting cell proliferation and triggering death of the tumor cells. Resveratrol has been widely described to have antioxidant and protective actions against a wide range of diseases, particularly cardiovascular diseases [15] and cancer [14]. However, other natural products obtained from the same source, the *Vitis vinifera* cane, in particular tetramers, have not been tested before for their antiproliferative activity against hepatoma cells. As resveratrol, some of these compounds, such as hopeaphenol, isohopeaphenol and R2-viniferin, were also identified in red wines and only recently R-viniferin and R2-viniferin could be detected at very low concentrations in certain red wines [20].

The dimer of resveratrol ε-viniferin was cytotoxic to various leukemia, HeLa cervix cancer, breast cancer, melanoma, and HepG2 cell lines [21]. The stilbene induced apoptosis in these cells and has been shown to inhibit topoisomerase IIa [22]. In HepG2, ε-viniferin showed slightly lower antiproliferative potential than resveratrol [23], and this was confirmed in the present work. The authors did not test toxicity in non-malignant liver cells. In our work, the resveratrol dimer also induced toxicity to HH4 cells, although the IC_50_ was high (178 µM). This result rules out the possibility to develop this product as a unique anticancer agent against hepatocellular carcinomas. However, the development of antitumor therapies could be established based on combinations of stilbenes at low concentrations with anticancer drugs, which may exert synergistic effects in the prevention or treatment of liver cancers, as it has been described for several cancer cells [24,25,26].

The results of this screening study showed that the cytotoxic effects of the natural stilbenes varied from one compound to another, and seemed to be dependent on the cellular model used. For example, in HepG2 hopeaphenol was two times more potent than its tetrameric geometric isomer isohopeaphenol. We do not know the cause of these differences in the cytotoxic activity of the two isomers. In a study reported by Loisruangsin et al. it was found that both tetramers acted as competitive inhibitors of sirtuin 1 (SIRT1), a key histone deacetylase in the regulation of cellular processes, suggesting that inhibitors could suppress the growth of tumor cells [27]. Hopeaphenol proved to be a more efficient inhibitor than isohopeaphenol. The authors showed, by computer-assisted modeling, that there were differences in the bonds that were established in the enzyme complex resulting from the inhibition, with more hydrogen bonds being formed in the case of hopeaphenol than with isohopeaphenol. As regards the different efficacy depending of the cell line, hopeaphenol and isohopeaphenol, similar to the resveratrol monomer, were more toxic to Hep3B than to HepG2. By contrast, R2-viniferin, a tetramer formed from two dimers of ε-viniferin and ampelopsin B, was the most potent stilbene to induce cell death in HepG2 (with an IC_50_ three times lower than that of resveratrol), while Hep3B cells were less sensitive to this tetramer. The structural diversity of tested compounds could explain the variability of activity on different cellular models. To better understand these structural specificities, additional studies will be necessary to look at the structure–activity relationship.

Scientific reports regarding the R2-viniferin stilbene are quite scarce. There is great confusion about this oligostilbene, since R2-viniferin is also called vitisin A, a name that also refers to another compound with a pyranoanthocyanin structure found in red wines [28]. Two studies reported that this oligostilbene induced cell death in prostate cancer [29] and leukemia cell lines [30]. In these latter cells, R2-viniferin induced cell apoptosis, as well as the inhibition of ERK, p38, and NF-kB pathways. In our work, the differences in susceptibility to R2-viniferin and the other resveratrol oligomers may be related to differences in the genetic background of the hepatocarcinoma cell lines. Thus, HepG2 cells carry wild-type p53, while Hep3B are p53-null. Tumor protein p53 induces cell cycle arrest and apoptosis through the transcriptional regulation of *BAX* gene. The protein also upregulates Bak expression, and the activation of Bax and Bak induces activation of caspase-3 [31]. In HepG2 cells, we have found that R2-viniferin tended to arrest cell cycle at G2/M phase, increased intracellular ROS levels, and the Bax/Bcl2 ratio in a dose-dependent manner. The induced effect on apoptosis is more dependent on the balance between Bcl-2 and Bax than on Bcl-2 quantity alone [32]. When tested at the same concentration of 10 µM, R2-viniferin was unable to inhibit cell proliferation and induce cell cycle blockage in p53-null Hep3B cells. In a previous work, resveratrol was reported to inhibit HepG2 cell proliferation by blocking cell cycle at the G2/M transition [33]. Since cyclins and their cyclin-dependent kinases (CDKs) are key regulators of cell cycle [34], the cell cycle inhibition by resveratrol in cancer cells has been attributed to disturbances in cyclins-CDKs complexes. We propose a similar mechanism of cell proliferation inhibition for R2-viniferin.

We have seen that R2-viniferin increased intracellular ROS levels without affecting the mitochondrial O_2_^−^ determined by the MitoSOX™Red fluorescent probe. We do not know the nature and source of these ROS. It has been described that resveratrol induces the expression of the transmembrane enzyme NADPH oxidase-5 (Nox5) in lung cancer cells. This enzyme generates O_2_^−^ which is converted by action of superoxide dismutase (SOD) into hydrogen peroxide [35]. In HepG2 resveratrol also causes the upregulation of SOD, without affecting glutathione peroxidase, which contributes to the formation and accumulation of hydrogen peroxide inside the cells [36]. Although speculative, R2-viniferin could exert similar effects in HepG2 in generating hydrogen peroxide, which would lead to cell apoptosis.

Our results show cytotoxic actions of stilbenes from *Vitis vinifera* cane in vitro. A major limitation of the study is the bioavailability of stilbenes in vivo. Several works have reported a weak bioavailability of resveratrol, mainly as a result of its low cell accessibility and its fast metabolism in the intestine and liver [37,38]. Therefore, a series of synthetic resveratrol derivatives that are more hydrophobic and with higher cell permeability are being developed to test their biological activity [39,40,41]. To our knowledge, there are no reports on the bioavailability of the stilbene oligomers screened in this study, with the exception of the dimer ε-viniferin [9,42,43], which showed by flow cytometry a cellular uptake kinetics similar to that of resveratrol [23]. The bioavailability of stilbenes depends on many factors, among them their stability, the molecular size, the chemical structure, and the hydrophilicity/hydrophobicity properties of the compound [44]. Encapsulation into nanoparticles or liposomes of bioactive compounds with low water solubility may be a promising approach to facilitate their stability, absorption, transport to target cells and, therefore, their action. This challenge has recently been described for ε-viniferin in Caco-2 intestinal cells [45].

## 5. Conclusions

We have described the cytotoxic activities of several natural resveratrol oligomers isolated from *Vitis vinifera* cane extracts against human hepatocellular carcinoma cells, and in comparison to HH4 non-transformed human hepatocytes. The cellular efficacies varied depending on the cell type. From the compounds tested, the tetramer R2-viniferin at concentrations below 10 µM was the most potent cytotoxic stilbene in p53-wilde type HepG2 cells, increasing intracellular ROS, and inducing cell apoptosis. The stilbene was innocuous in normal hepatocytes. These results suggest that R2-viniferin is a promising compound to develop in the chemoprevention and treatment of liver cancer. Further studies will be required in order to improve its bioavailability and to unravel its mechanism of action for potential clinical application in the treatment of liver cancer.

## Figures and Tables

**Table 1:** IC_50_^a^ values (µM) of different stilbenes against hepatocellular carcinoma cell lines.

Compound	Time (h)	HepG2	Hep3B	HH4
Monomer				
*trans*-Resveratrol	24 h	>200	>200	>200
	48 h	40.3 ± 9.3	42.0 ± 11.6	135.0 ± 9.0
	72 h	30.3 ± 4.4	21.0 ± 16.8	93.0 ± 16.1
Dimer				
Ampelopsin A	24 h	>200	>200	>200
	48 h	98.6 ± 24.9	147.8 ± 14.4	178.3 ± 67.8
	72 h	75.5 ± 21.5	109.1 ± 7.3	133.8 ± 34.7
*trans*-ε-Viniferin	24 h	140.0 ± 39.7	108.1 ± 31.8	>200
	48 h	103.7 ± 19.2	73.9 ± 17.3	192.7 ± 21.1
	72 h	94.8 ± 28.3	63.1 ± 10.8	177.9 ± 20.5
Tetramer				
Hopeaphenol	24 h	>200	>200	>200
	48 h	27.0 ± 3.3	16.8 ± 2.3	92.0 ± 38.0
	72 h	24.4 ± 2.0	13.1 ± 4.1	37.6 ± 13.0
Isohopeaphenol	24 h	113.0 ± 33.0	86.6 ± 11.7	>200
	48 h	68.8 ± 31.0	37.0 ± 4.5	96.0 ± 5.5
	72 h	54.1 ± 34.0	26.0 ± 3.0	63.7 ± 3.7
R2-Viniferin	24 h	>200	>200	>200
	48 h	10.2 ± 8.2	43.9 ± 3.6	>200
	72 h	9.7 ± 0.4	47.8 ± 2.8	>200
R-Viniferin	24 h	>200	>200	n.d ^b^
	48 h	>200	137.2 ± 19.8	n.d ^b^
	72 h	192.0 ± 27.1	134.9 ± 35.7	n.d ^b^

^a^ mean ± standard error; ^b^ n.d., not determined.

## Abbreviations

BSA	Bovine serum albumin
CDKs	Cyclin-dependent kinases
DCF	2´,7´-Dichlorofluorescein
DMSO	Dimethyl sulfoxide
DTT	Dithiothreitol
ECL	Enhanced chemiluminescence
EDTA	Ethylenediaminetetraacetic acid
EGTA	Ethylene glycol-bis(β-aminoethyl ether)-N,N,N′,N′-tetraacetic acid
ERK	Extracellular-signal-regulated kinase
GAPDH	Glyceraldehyde-3-phosphate dehydrogenase
H_2_DCF-DA	2′,7′ Dichlorodihydrofluorescein diacetate
HCC	Hepatocellular carcinoma
HEPES	4-(2-Hydroxyethyl)-1-piperazineethanesulfonic acid
NF-kB	Nuclear factor kappa B
O_2_^−^	Superoxide anion
PBS	Phosphate buffer saline
ROS	Reactive oxygen species
